# Reliability and psychometric properties of the Greek translation of the State-Trait Anxiety Inventory form Y: Preliminary data

**DOI:** 10.1186/1744-859X-5-2

**Published:** 2006-01-31

**Authors:** Konstantinos N Fountoulakis, Marina Papadopoulou, Soula Kleanthous, Anna Papadopoulou, Vasiliki Bizeli, Ioannis Nimatoudis, Apostolos Iacovides, George S Kaprinis

**Affiliations:** 1 3^rd ^Department of Psychiatry, Aristotle University of Thessaloniki, Greece

## Abstract

**Background:**

The State-Trait Anxiety Inventory form Y is a brief self-rating scale for the assessment of state and trait anxiety. The aim of the current preliminary study was to assess the psychometric properties of its Greek translation.

**Materials and methods:**

121 healthy volunteers 27.22 ± 10.61 years old, and 22 depressed patients 29.48 ± 9.28 years old entered the study. In 20 of them the instrument was re-applied 1–2 days later. Translation and Back Translation was made. The clinical diagnosis was reached with the SCAN v.2.0 and the IPDE. The Symptoms Rating Scale for Depression and Anxiety (SRSDA) and the EPQ were applied for cross-validation purposes. The Statistical Analysis included the Pearson Correlation Coefficient and the calculation of Cronbach's alpha.

**Results:**

The State score for healthy subjects was 34.30 ± 10.79 and the Trait score was 36.07 ± 10.47. The respected scores for the depressed patients were 56.22 ± 8.86 and 53.83 ± 10.87. Both State and Trait scores followed the normal distribution in control subjects. Cronbach's alpha was 0.93 for the State and 0.92 for the Trait subscale. The Pearson Correlation Coefficient between State and Trait subscales was 0.79. Both subscales correlated fairly with the anxiety subscale of the SRSDA. Test-retest reliability was excellent, with Pearson coefficient being between 0.75 and 0.98 for individual items and equal to 0.96 for State and 0.98 for Trait.

**Conclusion:**

The current study provided preliminary evidence concerning the reliability and the validity of the Greek translation of the STAI-form Y. Its properties are generally similar to those reported in the international literature, but further research is necessary.

## Background

The State-Trait Anxiety Inventory (STAI) – form Y is a brief self-rating scale for the assessment of state and trait anxiety, in adults. The concepts of state and trait anxiety were first introduced by Cattell [1-3] and have been further elaborated by Spielberger [4-7]

State anxiety (S-Anxiety) refers to the subjective and transitory feeling of tension, nervousness, worry and may be characterized by activation of the autonomous nervous system, at a given moment. Trait anxiety (T-Anxiety) refers to relatively stable individual differences in anxiety proneness as a personality trait, that is, in the tendency to perceive and respond to stressfull situations with elevations in the intensity of state anxiety (S-Anxiety) reactions.

In general, the STAI measures anxiety as a feature of the general population, thus it is expected its scores to follow the normal distribution. However it is widely used in the assessment of patient populations.

The State-Trait Anxiety Inventory (STAI) is reported to be reliable and valid and has been used extensively in research and clinical practice. The development of STAI was initiated in 1964 by C.D. Spielberger and R.L. Gorsuch and STAI-Form X was published in 1970 [8]. On the basis of accumulated knowledge gained from extensive research with the STAI, a revision of the scale began in 1979, and eventually Form Y was published in 1985. The STAI comprises separate self-report scales for measuring state and trait anxiety, consistent with the definitions given above. The S-Anxiety scale (STAI Form Y-1) consists of twenty statements that evaluate how the respondent feels "right now, at this moment". The T- Anxiety scale (STAI Form Y-2) consists of twenty statements that evaluate how the respondent feels "generally". In responding to the S-Anxiety scale, the subjects choose the number that best describes the intensity of their feelings: (1) not at all, (2) somewhat, (3) moderately, (4) very much so. In responding to the T-Anxiety scale, subjects rate the frequency of their feelings on the following four-point scale: (1) almost never, (2) sometimes, (3) often, (4) almost always. Each STAI item is given a weighted score of 1 to 4.

A rating of 4 indicates the presence of high levels of anxiety for ten S-Anxiety items (#3, 4, 6, 7, 9, 12, 13, 14, 17 and 18) and eleven T-Anxiety items (#22, 24, 25, 28, 29, 31, 32, 35, 37, 38, 40). A high rating indicates the absence of anxiety for the remaining ten S-Anxiety items and nine T-Anxiety items. The scoring weights for the anxiety-present items are the same as the chosen numbers on the test form. The scoring weights for the anxiety-absent items are reversed. Scores for both the S-Anxiety and the T-Anxiety scales can vary from a minimum of 20 to a maximum of 80.

The aim of the current preliminary study was to assess the reliability and the psychometric properties of the Greek translation of the State-Trait Anxiety Inventory (STAI) – form Y.

## Materials and methods

The present study included 121 healthy volunteers aged 27.22 ± 10.61 years old, and 22 depressed patients aged 29.48 ± 9.28 years old.

This mixed population was chosen because of the nature of the instrument. The STAI principally measures anxiety as a feature of the general population, so the main study sample to test the properties of the instrument should be 'healthy normal subjects'. However it is also important to test the properties of the instrument in a population that manifests higher than normal levels of anxiety. Depressed patients were chosen on the basis that this patients population was easier for the researchers to recruit taking into consideration practical issues.

Patients were physically healthy with normal clinical and laboratory findings (Electroencephalogram, blood and biochemical testing, thyroid function, test for pregnancy, B12 and folic acid). They came from the inpatient and outpatient unit of the 3^rd ^Department of Psychiatry, Aristotle University of Thessaloniki, General Hospital AHEPA, Thessaloniki, Greece. They were consecutive cases and were chosen because they fulfilled the above criteria.

The normal controls group was composed by members of the hospital staff, students and other volunteers. A clinical interview confirmed that they did not suffer from any mental disorder and their prior history was free from mental and thyroid disorder. They were free of any medication for at least two weeks and were physically healthy.

All patients and controls provided written informed consent before participating in the study.

**Translation and back translation **were made by two of the authors; one of whom did the translation and the other who did not know the original English text did the back translation. The final translation was fixed by consensus of all authors.

The Greek translation along with the translated manual of the test will be available from the same publisher of the English version of the test and manual.

**Clinical diagnosis **was reached with the Schedules for Clinical Assessment in Neuropsychiatry (SCAN) version 2.0 [9,10] and the International Personality Disorders Examination (IPDE) [11-14]. Both were applied by one of the authors (KNF) who has official training in a World Health Organization Training and Reference Center. The IPDE did not contribute to the clinical diagnosis of anxiety and/or depression, but was used in the frame of a global and comprehensive assessment of the patients. The second examiner performed an unstructured interview. The Symptoms Rating Scale for Depression and Anxiety (SRSDA) which provides an Anxiety index and a Beck Depression Inventory-21 score and the Eysenk Personality Questionnaire (EPQ) were applied for cross-validation purposes.

In 20 of the patients the instrument was re-applied 1–2 days later

### Statistical analysis

Analysis of Variance (ANOVA)[15], was used to search for differences between groups, with Scheffe test as the post-hoc test.

**Figure 1:**
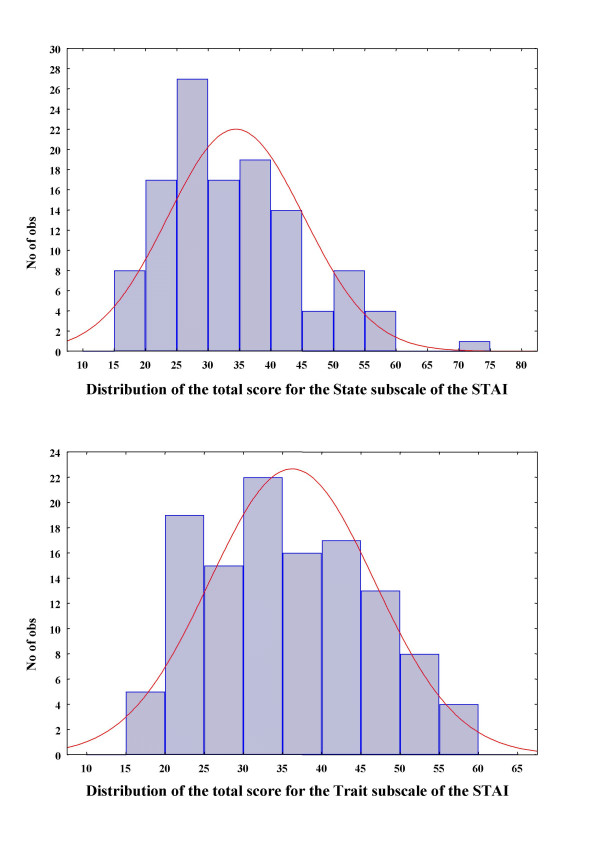
Distribution of the Trait and State scores of the STAI in normal subjects. State does not follow the normal distribution, on the contrast Trait follows it

The Kolmogorov-Smirnov test was used to test whether the State and Trait subscales follow the normal distribution in normal subjects (figure 1).

Principal Components Analysis (without and after Varimax Normalized Rotation) was performed, and factor coefficients and scores were calculated.

Item Analysis [16] was performed, and the value of Cronbach's alpha (α) for each STAI subscale was calculated.

During both Principal Components Analysis and Item Analysis, all subjects (both normal volunteers and depressed patients) were included and all the items scores were turned to the direction of the presence of anxiety. Principal Component Analysis was performed also with the inclusion of normal subjects alone.

### Reliability assessment (test-retest)

The Spearman Rank Correlation Coefficient (rho) was calculated to assess the test-retest reliability. However, the calculation of correlation coefficients is not a sufficient method to test reliability and reproducibility of a method and its results, because it is an index of correlation and not an index of agreement [15,17,18]. The calculation of means and standard deviations for each STAI item and total score during the 1^st ^(test) and 2^nd ^(retest) applications may provide an impression of the stability of results over time.

**Figure 2:**
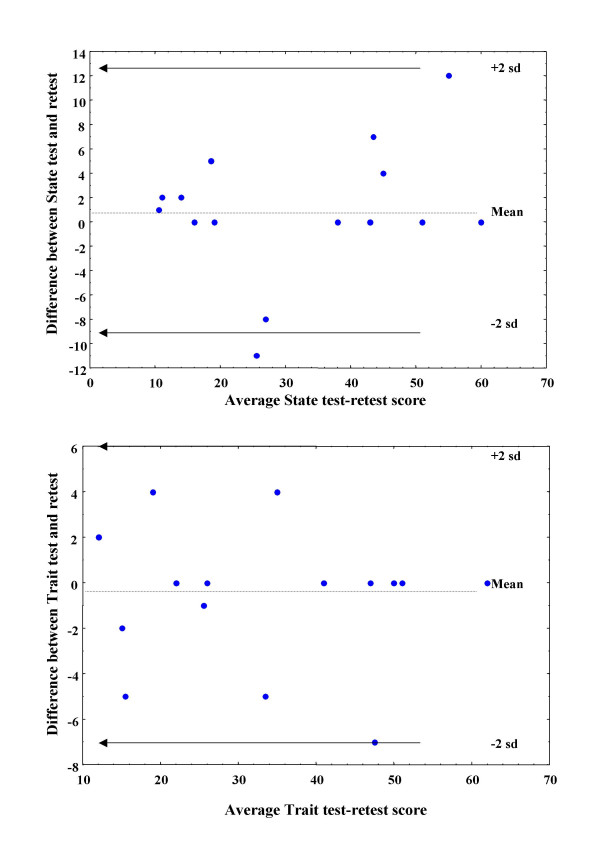
Axis x: average (test and retest) State and Trait STAI scales scores. Axis y: the mean difference concerning the State and Trait STAI scales between test and retest

Also, the means and the standard deviations of the differences concerning each STAI item between test and retest were calculated and the plots of the test vs. retest and difference vs. average value for each variable were created. In fact it is not possible to use statistics to define acceptable agreement [15]. However these plots may assist decision. It is not possible to show all of these plots, but the respected concerning the STAI State and Trait Scores is shown in figures 2. This method was used in previous studies concerning the validation of scientific methods [19,20].

Also, the module of 'Process Analysis Gage Repeatability and Reproducibility' of the Statsoft-Statistica was used to further investigate the repeatability of the STAI with the use of Analysis of Variance (ANOVA) [21]. The purpose of this analysis is to determine the proportion of measurement variability that is due to

1. the subjects being assessed

2. the STAI items (method) used for the measurement

3. the trials (in our case: test vs. retest)

In the ideal case, only a negligible proportion of the variability will be due to trial-to-trial repeatability.

## Results

The State score for the healthy volunteers was 24.95 ± 11.36 and the Trait score was 27.88 ± 11.43. The respected scores for the depressed patients were 44.91 ± 9.18 and 43.50 ± 9.99 respectively (F = 55.58, df = 2, p < 0.001; post-hoc Scheffe test: p < 0.001 for both State and Trait subscales). The Kolmogorov-Smirnov test with the use of normal subjects alone, revealed that State does not follow the normal distribution (p < 0.001); on the contrary, Trait follows the normal distribution (p = 0.15; figure 1).

Cronbach's alpha was 0.93 for the State and 0.92 for the Trait subscale. The Spearman Rank Correlation Coefficient between State and Trait subscales was 0.79. Both subscales correlated strongly with the anxiety subscale of the SRSDA, but also with the Beck Depression Inventory-21 and the EPQ dimensions (table 1)

**Table 1:** Correlations of State and Trait subscales of the STAI to other psychometric scales

	**State**	**Trait**
**Age**	-0.08	-0.11
**Years of education**	-0.21	-0.21
**Anxiety subscale of the SRSDA**	0.79	0.72
**BDI-21**	0.75	0.73
**EPQ P**	0.22	0.26
**EPQ N**	0.60	0.70
**EPQ E**	-0.35	-0.48
**EPQ L**	-0.13	-0.18

The test-retest reliability was excellent, with Pearson coefficient being between 0.75 and 0.98 for individual items and equal to 0.96 for State and 0.98 for Trait (table 2). The descriptive statistics of test vs. retest are shown in table 3. The bivariate scatterplots of the differences between measurements vs. the average value of measurements concerning the State and Trait scores (figure 2) suggest that both the State and the Trait subscales are reliable, since almost all the points of the difference vs. average are within the 2 standard deviation range from the mean difference. The Process Analysis Gage Repeatability and Reproducibility revealed that the proportion of variance due to test-retest variability is negligible.

**Table 2:** Test-retest correlation coefficients for each STAI item separately and for State and Trait total scores

	**Pearson's R**		**Pearson's R**
**Anxiety subscale of the SRSDA**	0.98		
**State**		**Trait**	

**State item no 1**	0.94	**Trait item no 21**	0.94
**State item no 2**	0.90	**Trait item no 22**	0.94
**State item no 3**	0.92	**Trait item no 23**	0.92
**State item no 4**	0.84	**Trait item no 24**	0.84
**State item no 5**	0.85	**Trait item no 25**	0.93
**State item no 6**	0.95	**Trait item no 26**	0.78
**State item no 7**	0.98	**Trait item no 27**	0.91
**State item no 8**	0.98	**Trait item no 28**	0.94
**State item no 9**	0.96	**Trait item no 29**	0.97
**State item no 10**	0.86	**Trait item no 30**	0.91
**State item no 11**	0.95	**Trait item no 31**	0.97
**State item no 12**	0.94	**Trait item no 32**	0.98
**State item no 13**	0.94	**Trait item no 33**	0.98
**State item no 14**	0.89	**Trait item no 34**	0.96
**State item no 15**	0.93	**Trait item no 35**	0.93
**State item no 16**	0.97	**Trait item no 36**	0.92
**State item no 17**	0.89	**Trait item no 37**	0.83
**State item no 18**	0.91	**Trait item no 38**	0.95
**State item no 19**	0.87	**Trait item no 39**	0.75
**State item no 20**	0.84	**Trait item no 40**	0.83
**State total score**	0.96	**Trait total score**	0.98

**Table 3:** Descriptive statistics of test and retest applications of the STAI

	**Mean**	**Std.Dev.**
**State test**	34.00	18.64
**State retest**	32.78	16.48
**Trait test**	31.88	15.86
**Trait retest**	32.83	15.42
**State difference test minus retest**	0.88	5.26
**Trait difference test minus retest**	-0.50	3.06

The Principal Components Analysis (varimax normalized rotation) with the use of the total study sample revealed the presence of 7 factors explaining 69% of the total variance (table 3). The use of normal subjects alone produced similar results. Three of these factors were stronger, explaining 18%, 15% and 13%, that is two thirds of explained variance. Factor 1 corresponds to a well-being factor, factor 3 to trait and factor 4 to state anxiety. The rest four factors are rather residual factors corresponding to less strong aspects of positive (factor 2) or negative (factor 5) sense of well-being, or to various negative cognitive processes like insecurity, worries, lack of self-confidence etc. (factors 6 and 7).

Items #7 and #17 from the state subscale and #27 from the trait subscale load both on the trait and state factors, and this puts in question their ability to distinguish between state and trait anxiety.

## Discussion

The results of the current validation study suggest that the Greek translation of the STAI is both reliable and valid, with psychometric properties close to those reported in the international literature. However, the mean scores for normal subjects were substantially lower than those reported in the English STAI Manual [22] (State 24.95 ± 11.36 vs. 36.54 ± 10.22 and the Trait score was 27.88 ± 11.43 vs. 35.55 ± 9.76). The factor analysis results are generally in accord with the literature, and support both the state-trait distinction but also the presence of a well being dimension.

There are several translations of the STAI in various languages around the world. It seems these translations may manifest different properties one from another, however, all have high reliability.

Cronbach's alpha for the Dutch translation of the short-form of the STAI-state was 0.83. The short form highly correlates with the full form (r = 0.95) [23]. The Malaysian version of the STAI has Cronbach's alpha value = 0.86 and high test-retest reliability and sensitivity to treatment [24]. The testing of the psychometric properties of four self-report anxiety measures including the STAI revealed an adequate internal consistency for all measures. Test-retest reliability over a 2–4 week interval was mixed, with some measures apparently assessing stable, trait-like dimensions of fear and anxiety, and others estimating more state-like clinical features [25].

As measured by Jackson's (1970) Differential Reliability Index, content saturation was found to be high for only 7 A-State and A-Trait items [26]. Thus, there was an effort to produce shorter STAI versions. A 5-item short form of the STAI is reported to have optimal reliability and validity, and also a balance of items from the Worry and Emotionality subscales [27]. Also, a 6-item version of the STAI is reported to have high correlation with the full scale score and acceptable reliability and validity [28]. Additionally, several items of the STAI produce misfit responses and do not produce equal units of measurement. These findings question the generalizability of the research on anxiety [29].

Another question concerns the reliability of the instrument when applied to special populations, especially the elderly. The STAI scale demonstrated high internal reliability when applied to elderly subjects [30].

The validity of the instrument when applied to elderly patients is another question. A conspicuously high score on the state part of the STAI has been observed among geriatric inpatients which were neither demented nor critically ill; 43% of them had a score that, according to Spielberger's criteria, would reflect clinically relevant anxiety symptoms. High item-scores were more frequent on the symptom-negative items than on the symptom-positive items. The most probable explanation is that the STAI State score is a biased indicator of anxiety in geriatric inpatients owing to confounding by reduced well-being in these patients [31].

The validation of the Portuguese version of the STAI reported mean scores for anxious patients equal to 52.8 ± 11.4 and for depressed patients equal to 56.4 ± 10.5, and higher than for the student sample which was 40.7 ± 8.6 [32]. These results are different both from ours and from the US data, and normal students data are much higher than expected. Several factors are reported to influence the STAI score. Trait scores are reported to be higher for women, singles, those who work, and subjects under 30 years old [33]. Apparent ethnic differences in anxiety levels may be due to causal variables related to other sociodemographic variables. Thus, using only a global STAI composite as a measure of anxiety will mask the differential effects of the STAI factor scores [34]. For example, although mean scores for the state and trait anxiety-absent items were comparable for Japanese students living in Japan and Japanese international students studying in the United States, the scores of both Japanese groups were significantly higher than those of American students. These differences were attributable to much higher scores of Japanese students on anxiety-absent items that corresponded to a lack of positive feelings. Japanese students had a tendency to inhibit positive (anxiety-absent) feelings, resulting in higher anxiety scores [35]. Also, the mean STAI State and Trait anxiety scores of Japanese workers were substantially higher than those of American workers reported in the Manual, due primarily to the much higher scores of Japanese workers in responding to the anxiety-absent items. The correlations between the State and Trait anxiety-present scales and those of their anxiety-absent scales' counterparts were higher than those between the State anxiety-present and -absent scales and those of their Trait scales' counterparts. These findings suggested that responses to anxiety-present and -absent items should be considered independently in scoring the STAI scales in Japanese working adults [36].

An additional problem concerning the STAI scales validity is the fact that several researchers have found anxiety and depression to be indistinguishable in non-clinical samples and have suggested that both constructs may be components of a general psychological distress process [37]. So, STAI may in fact measure this non-specific distress and not pure anxiety per se. In accord with this is the report that in geriatric inpatients who are neither demented nor critically ill, multi-group factor analysis produced two factors termed 'well-being' and 'nervousness', which had a moderate correlation (0.61)[31].

Factor analytic studies produce mixed results, others in favor and others against the state-trait distinction. The factor analysis of the Japanese STAI extracted 3 factors, 'anxiety-absent', 'state anxiety-present' and 'trait anxiety-present' [38]. This analysis suggests that the three components were considered to reflect the "overall anxiety" component, the "presence-absence of anxiety (positive-negative)" component, and the "state-trait anxiety" component. The component related to presence or absence of anxiety was larger than the state-trait anxiety component [39]. Confirmatory factor analytic methods suggested that a hierarchical solution could best fit the data, with one overall factor and two lower order factors. This paper supports the notion that the trait scale of the STAI assesses depression, as well as anxiety. One set of items appeared to assess anxiety and worry, whereas the other assessed sadness and self-deprecation. The two subscales correlated differentially with other measures of anxiety and depression in a manner that was consistent with their content [40]. The STAI scale when applied to elderly subjects did not manifested factorial validity, with analyses failing to support presence of state and trait anxiety factors [30].

Other factor analytic studies have provided support for the concepts of state and trait anxiety. Some authors suggest that a two-factor state vs. trait solution is the most appropriate, accounting for 41.1% of the variance [41]. Others propose a solution with 2 trait factors and 4 transient sources of true variance [42]. In a sample from Hawaii, a four-factor model (State-Anxiety Absent, State-Anxiety Present, Trait-Anxiety Absent, and Trait-Anxiety Present) provided the best fit [43]. Another factor analysis of the Japanese STAI produced different results in contrast to previous studies [38,39] and reported a 4-factor solution (positively and negatively worded state factors, positively and negatively worded trait factors)[44], which is in accord with the state-trait distinction.

## Conclusion

The current study provided preliminary evidence concerning the reliability and the validity of the Greek translation of the STAI-form Y. Its properties are generally similar to those reported in the international literature, but further research is necessary.

**Table 4:** Factor analysis of State and Trait STAI items. All values >0.35 are in bold underlined fonts.

	**Factor 1**	**Factor 2**	**Factor 3**	**Factor 4**	**Factor 5**	**Factor 6**	**Factor 7**
**State item no 1**	0.35	0.01	-0.12	***0.69***	-0.10	-0.11	0.21
**State item no 2**	0.22	0.04	-0.23	***-0.52***	-0.11	***-0.47***	-0.13
**State item no 3**	-0.18	0.02	0.31	***0.75***	0.02	-0.15	-0.13
**State item no 4**	-0.04	0.02	0.35	***0.73***	0.08	0.20	-0.09
**State item no 5**	***0.40***	0.03	-0.15	***-0.67***	-0.04	-0.29	-0.07
**State item no 6**	-0.22	0.00	0.31	***0.61***	***0.37***	0.07	-0.09
**State item no 7**	-0.12	-0.05	***0.41***	***0.49***	0.04	0.15	***0.49***
**State item no 8**	***0.56***	0.18	0.04	***-0.43***	-0.20	-0.21	-0.15
**State item no 9**	0.02	-0.14	0.30	***0.48***	0.33	0.22	0.23
**State item no 10**	***0.53***	-0.06	-0.03	***-0.65***	-0.03	-0.19	-0.11
**State item no 11**	***0.37***	-0.04	-0.14	-0.29	-0.14	***-0.71***	0.05
**State item no 12**	-0.29	0.02	0.32	***0.63***	0.30	0.03	-0.09
**State item no 13**	0.19	***-0.80***	0.16	-0.15	-0.07	0.05	0.04
**State item no 14**	-0.08	-0.01	0.15	0.26	***0.76***	0.27	0.01
**State item no 15**	***0.47***	0.01	-0.19	***-0.64***	-0.23	-0.14	0.08
**State item no 16**	***0.61***	0.02	-0.22	***-0.52***	-0.01	-0.25	-0.04
**State item no 17**	-0.23	0.00	***0.42***	***0.64***	0.17	0.12	0.27
**State item no 18**	-0.20	0.08	0.25	***0.60***	***0.37***	0.17	-0.03
**State item no 19**	0.06	***0.84***	-0.10	-0.08	0.04	-0.18	0.01
**State item no 20**	***0.60***	-0.01	-0.28	***-0.56***	-0.03	-0.14	0.02
**Trait item no 21**	***0.76***	0.07	-0.34	-0.27	0.01	-0.19	0.03
**Trait item no 22**	***-0.37***	-0.20	***0.48***	0.30	0.15	0.12	-0.08
**Trait item no 23**	-0.08	***0.88***	-0.16	0.08	-0.04	-0.01	0.10
**Trait item no 24**	-0.08	0.00	***0.62***	0.14	0.10	0.22	-0.13
**Trait item no 25**	-0.10	-0.05	***0.56***	0.06	0.20	***0.49***	-0.22
**Trait item no 26**	***0.70***	-0.09	-0.18	-0.16	-0.11	-0.05	-0.07
**Trait item no 27**	***0.51***	0.05	***-0.41***	***-0.41***	-0.05	-0.32	0.17
**Trait item no 28**	***-0.43***	0.03	***0.68***	0.13	0.26	0.19	0.01
**Trait item no 29**	-0.06	-0.09	***0.74***	0.24	0.10	0.05	-0.14
**Trait item no 30**	0.29	***0.83***	0.02	-0.14	0.09	0.02	0.02
**Trait item no 31**	-0.32	-0.12	***0.72***	0.26	0.04	0.19	0.09
**Trait item no 32**	-0.25	-0.03	***0.49***	0.14	0.25	***0.58***	-0.06
**Trait item no 33**	***0.43***	0.09	-0.32	-0.24	-0.19	***-0.45***	0.20
**Trait item no 34**	0.07	***0.74***	0.05	0.02	-0.27	0.18	0.01
**Trait item no 35**	-0.30	-0.07	0.35	0.25	***0.68***	0.07	-0.13
**Trait item no 36**	***0.71***	0.11	-0.29	-0.21	-0.20	-0.07	0.20
**Trait item no 37**	-0.09	-0.17	***0.71***	0.35	-0.10	0.05	0.06
**Trait item no 38**	-0.26	-0.10	***0.76***	0.22	0.16	0.01	0.05
**Trait item no 39**	0.13	0.18	-0.24	-0.29	-0.13	-0.29	***0.66***
**Trait item no 40**	-0.23	-0.02	***0.60***	0.34	0.25	0.01	0.25
**Expl.Var**	5.17	3.62	6.11	7.18	2.20	2.48	1.27
**Prp.Totl**	13%	9%	15%	18%	5%	6%	3%
**Total var expl**							69%
